# Advancing *Eucalyptus *genomics: identification and sequencing of lignin biosynthesis genes from deep-coverage BAC libraries

**DOI:** 10.1186/1471-2164-12-137

**Published:** 2011-03-04

**Authors:** Jorge AP Paiva, Elisa Prat, Sonia Vautrin, Mauro D Santos, Hélène San-Clemente, Sérgio Brommonschenkel, Paulo GS Fonseca, Dario Grattapaglia, Xiang Song, Jetty SS Ammiraju, David Kudrna, Rod A Wing, Ana T Freitas, Hélène Bergès, Jacqueline Grima-Pettenati

**Affiliations:** 1 Instituto de Investigação Científica Tropical (IICT), Centro de Florestas e dos Produtos Florestais, Tapada da Ajuda, 1349-018 Lisboa, Portugal; 2 Instituto de Biologia Experimental e Tecnológica, Apartado 12, 2781-901 Oeiras, Portugal; 3 INRA-CNRGV, Chemin de Borde Rouge, 31326 Castanet-Tolosan, France; 4 Instituto de Engenharia de Sistemas e Computadores: Investigação e Desenvolvimento (INESC-ID/IST), R. Alves Redol 9, 1000-029 Lisboa, Portugal; 5 Université de Toulouse; UPS; UMR 5546, Surfaces Cellulaires et Signalisation chez les Végétaux; BP 42617, F-31326, Castanet-Tolosan, France; 6 CNRS; UMR 5546; BP 42617, F-31326, Castanet-Tolosan, France; 7 BIOAGRO - Federal University of Viçosa, Av. P. H. Rolfs, s/n - 36570-000 - Viçosa, MG, Brasil; 8 EMBRAPA Genetic Resources and Biotechnology, EPqB Final W5 NOrte, 70770-910 Brasilia, DF, Brazil; 9 Arizona Genomics Institute, School of Plant Sciences and BIO5 Institute, The University of Arizona, Tucson AZ 85721, USA

## Abstract

**Background:**

*Eucalyptus *species are among the most planted hardwoods in the world because of their rapid growth, adaptability and valuable wood properties. The development and integration of genomic resources into breeding practice will be increasingly important in the decades to come. Bacterial artificial chromosome (BAC) libraries are key genomic tools that enable positional cloning of important traits, synteny evaluation, and the development of genome framework physical maps for genetic linkage and genome sequencing.

**Results:**

We describe the construction and characterization of two deep-coverage BAC libraries EG_Ba and EG_Bb obtained from nuclear DNA fragments of *E. grandis *(clone BRASUZ1) digested with *Hind*III and *BstY*I, respectively. Genome coverages of 17 and 15 haploid genome equivalents were estimated for EG_Ba and EG_Bb, respectively. Both libraries contained large inserts, with average sizes ranging from 135 Kb (Eg_Bb) to 157 Kb (Eg_Ba), very low extra-nuclear genome contamination providing a probability of finding a single copy gene ≥ 99.99%. Libraries were screened for the presence of several genes of interest *via *hybridizations to high-density BAC filters followed by PCR validation. Five selected BAC clones were sequenced and assembled using the Roche GS FLX technology providing the whole sequence of the *E. grandis *chloroplast genome, and complete genomic sequences of important lignin biosynthesis genes.

**Conclusions:**

The two *E. grandis *BAC libraries described in this study represent an important milestone for the advancement of *Eucalyptus *genomics and forest tree research. These BAC resources have a highly redundant genome coverage (> 15×), contain large average inserts and have a very low percentage of clones with organellar DNA or empty vectors. These publicly available BAC libraries are thus suitable for a broad range of applications in genetic and genomic research in *Eucalyptus *and possibly in related species of *Myrtaceae*, including genome sequencing, gene isolation, functional and comparative genomics. Because they have been constructed using the same tree (*E. grandis *BRASUZ1) whose full genome is being sequenced, they should prove instrumental for assembly and gap filling of the upcoming *Eucalyptus *reference genome sequence.

## Background

Renowned for their fast growth, valuable wood properties and wide adaptability, *Eucalyptus *species are among the most planted hardwoods in the world. The genus *Eucalyptus *includes over 700 species [1], including the most planted species *E. grandis *and *E. urophylla *(section *Transversaria*), *E. globulus *(section *Maidenaria*) and *E. camaldulensis *(section *Exsertaria*), all belonging to the subgenus *Symphyomyrtus*. Native to Australia, these fast growing trees were rapidly introduced into India, France, Chile, Brazil, South Africa, and Portugal in the first quarter of the 1800 s [2] and were promptly adopted for plantation forestry. Nowadays, *Eucalyptus *and their hybrids are among the world's leading sources of woody biomass and are the main hardwoods used for pulpwood and timber. In particular, *E. grandis *is grown in tropical and subtropical regions and *E. globulus *in limited temperate regions of the world while their hybrids are among the most widely used for industrial plantations because of their rapid growth rate, their adaptability to diverse ecological conditions and their good quality wood fiber.

Because of their high commercial value, *Eucalyptus *species are major targets for genetic improvement. Nevertheless from a genetics perspective, *Eucalyptus *are still in the early stages of domestication when compared to crop species and this fact has important implications when applying genomics approaches to understand the structural and functional biology of the genome [3-5].

In the last 15 years, several studies have led to a better understanding of the *Eucalyptus *genome and the development of an important set of genetic/genomic tools, which can be used to enhance future breeding efforts. *Eucalyptus *sp. are diploid plants with a haploid chromosome number of 11 [6-8]. Grattapaglia and Bradshaw [8] estimated the haploid genome size of Eucalyptus species to range ranging from 370 to 700 million base pairs (Mbp), with the *Symphyomyrtus *species having on average a haploid genome size of 650 Mbp. The high level of genetic diversity, the ability to generate large progeny sets, the relatively small genome size and low proportion of repetitive DNA facilitated genetic linkage mapping in *Eucalyptus *[9]. Most *Eucalyptus *linkage maps [3-5], have been constructed from highly heterozygous parents and segregating half-sib of full-sib families, with progenies up to 200 individuals often from different species. The development of co-dominant markers such as microsatellites allowed comparative mapping studies among *Eucalyptus *species. Extensive map synteny and co linearity were detected between species of the subgenus *Symphomyrtus *[10]. QTL mapping in *Eucalyptus *has been applied to the identification of genetic loci associated with variation in biomass productivity, stem form, wood properties (wood density and composition, fiber traits, bark composition), vegetative propagation, biotic/abiotic stress responses, development, foliar chemistry, inbreeding depression, and transcript level [4,5]. Expressed sequence tag (EST) catalogues are available [11-16] and private consortia generated thousands of other ESTs. The complete nucleotide sequence of the chloroplast genome from *E. globulus *is also available [17], and is similar to other angiosperms, with an inverted repeat (IR) separated by a large single copy (LCS) region, and a small single copy (SSC) region.

Currently in progress, the first draft of a whole genome shotgun assembly of the *Eucalyptus *genome (*E. grandis*, clone BRASUZ1; http://www.eucagen.org) is expected to be publicly available by mid 2010. This effort will represent a major achievement and can be used as the first *Eucalyptus *reference sequence for future genomic undertakings [5].

Bacterial artificial chromosome (BAC) libraries have served as an essential genomic tool to enable positional cloning of important traits, synteny evaluation, and the development of genome framework physical maps for genetic linkage and genome sequencing [18]. A number of BAC libraries have been reported for woody plants, including forest trees such as *Liriodendron tulipifera *[19], *Pinus pinaster *[20,21], *Populus trichocarpa *[22,23] and *P. tremuloides *[23,24]. The first *E. grandis *BAC library was built in Brazil in the context of the Genolyptus project, supplying full genomic clones of key genes as well as end-sequences used to derive microsatellite and STS markers to help anchoring selected BAC clones to the existing linkage maps [25]. Unfortunately, due to intellectual property restrictions, this library in not publicly available and no detailed report has been published yet about this specific work.

In this study, we report the construction and characterization of two deep-coverage BAC libraries (15-17×) from *E. grandis *(genotype "BRASUZ1") whose genome is currently being sequenced, becoming the public reference genome for the genus *Eucalyptus *http://www.eucagen.org. The libraries described here i.e. EG_Ba (constructed from *Hind*III restricted nuclear DNA fragments) and EG_Bb library (constructed from *Bst*YI-digested DNA), are the first large-insert DNA libraries publicly available for genus *Eucalyptus *http://www.genome.arizona.edu/orders/. The Roche GS FLX technology was successfully used to sequence five BAC clones allowing us to report here the whole sequence of the *E. grandis *chloroplast genome as well as the genomic sequences of important lignin biosynthetic genes.

## Results and discussion

### BAC library characterization

Two genomic BAC libraries (EG_Ba and EG_Bb) were constructed using partially digested (*Hind*III or *BstY*I) and size-selected nuclear DNA isolated from *E. grandis *(genotype BRASUZ1) and the pAGIBAC1 cloning vector, as described in Materials and Methods. Two libraries were constructed, using different restriction enzymes to avoid a biased distribution of clones along the *Eucalyptus *genome [26-29]. Each library, EG_Ba (*Hind*III) and EG_Bb (*BstY*I), contains 73,728 robotically picked clones arrayed into 192 384-well microtiter plates.

**Figure 1:**
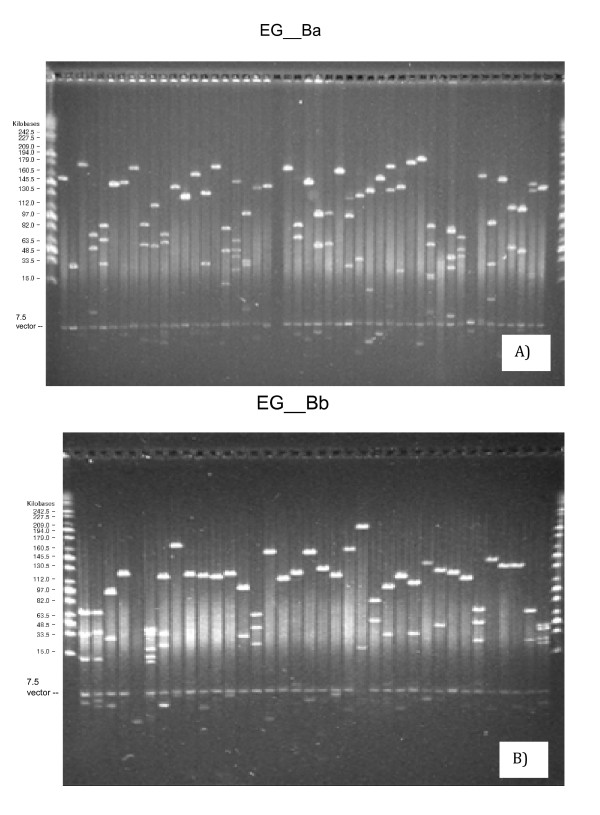
**NotI digest of random *E. grandis *BAC clones**. PFGE random selected BAC clones from the a) EG__Ba and b) EG__Bb libraries. Size standards and cloning vector are indicated.

**Figure 2:**
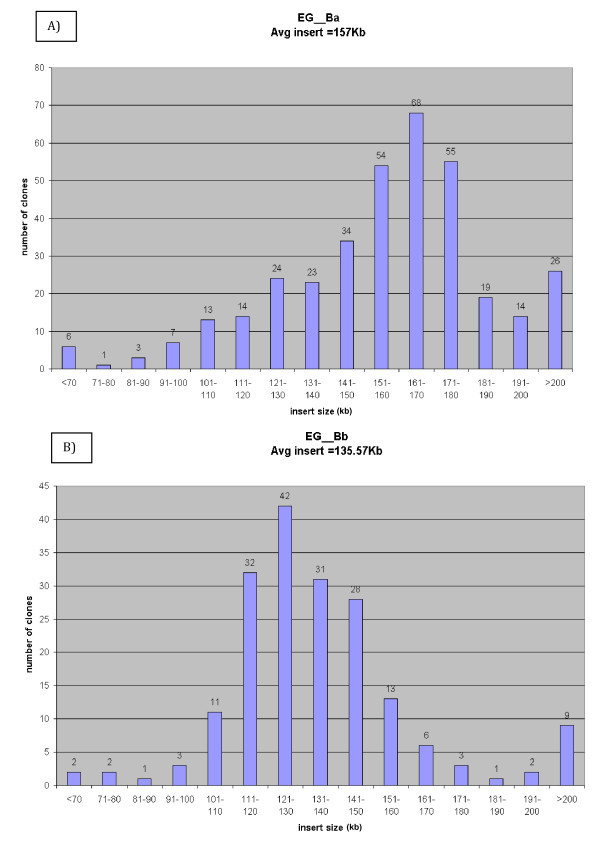
**Size distribution of the inserts in the two BAC libraries**. Histogram shows the distribution of insert sizes from random selected BAC clones from the a) EG__Ba BAC library and the b) EG__Bb BAC library.

To evaluate the average insert size of each library, BAC DNA was isolated about 384 randomly selected clones from each library, restriction enzyme digested with the rare cutter *Not*I, and analyzed by Pulsed-Field Gel Electrophoresis (PFGE). All fragments generated by *Not*I digestion contained the 7.5 kb vector band and various insert fragments (see Figure 1a, b). Analysis of the insert sizes from the EG_Ba library showed that more than 87% of the library contained inserts >120 kb while the average insert size was 157 kb (Figure 2a). Analysis of the insert sizes from the EG_Bb library showed that more than 89% of the library contained inserts >111 kb while the average insert size was 135 kb (Figure 2b). Since the haploid genome of *E. grandis *is about 660 Mb, the library coverage is predicted to be 17 and 15 haploid genome equivalents for the EG_Ba and EG_Bb libraries, respectively: large large enough coverage to ensure these libraries will be useful for positional cloning, physical mapping and genome sequencing [30]. The estimated probability of finding any specific sequence is greater than 99.99% considering both libraries together [31].

### BAC library screening

To characterize these BAC libraries and facilitate clone identification, we prepared high-density macroarrays on nylon filters from a subset of the libraries representing 8.5× and 7.5× of genome coverage for EG_Ba and EG_Bb libraries, respectively. Membranes were hybridized with a series of pooled probes representing the chloroplast and mitochondria genome as well as with probes derived from lignin biosynthesis related genes.

Contamination with extranuclear genomes was estimated at 0.55% of the total number of BAC clones in both libraries. BAC clones containing *E. grandis *chloroplast sequences represented about 0.48% of all BAC clones in both libraries, lower than the estimates for several other plant species libraries [32-37]. The mitochondrial genome was represented by 0.07% of our BAC clones; slightly higher as compared to the 0.03%, 0.012%, and 0.04% found in coffee [33], tomato [38], and banana [39] BAC libraries, respectively.

To evaluate the potential of these two BAC libraries to supply genomic sequences to contain candidate genes for cell wall biosynthesis, we screened the libraries with cDNA probes for lignin biosynthesis genes, [14,40-42] and regulatory genes (*EgMyb1*, *EgMyb2*, *EgRAC1*) [11,43,44]. An average of 15.6 positive clones (Table 1) was obtained when the EG_Ba library was screened with probes derived from the following genes: *EguCAD2 *(22 clones), *EguCCoAOMT *(19 clones), *EguCCR1 *(13 clones), *EguF5 H *(17 clones), *EguHCT *(13 clones), *EguMyb1 *(10 clones) and *EguPAL *(15 clones). An average of 14.2 positive clones was obtained by probing the EG_Bb macroarray against *Egu4CL *(15 clones), *EguC3 H *(5 clones), *EguC4 H *(6 clones), *EguMyb2 *(7 clones), *EguCOMT *(19 clones) and *EguRAC1 *(33 clones) genes. The results of the macroarray hybridization gene screening suggest an over-representation of positive clones for *CAD2, CCoAOMT, COMT *and *RAC *genes. However, the probes used in the macroarray hybridizations were relatively long allowing the possibility of cross-hybridization with other members of multigene families.

**Table 1:** BAC libraries screening. cDNA probes used were either involved in the lignin biosynthetic pathway (*PAL, C4 H, HCT, C3 H, CCoAOMT, CCR, F5H/CAld5H*, *CAD*) or regulating this pathway (*MYB1 *and *2*, *RAC1*).

Gene family	Gene	EMBL accession	library	Positive clones Array hybridization (a)	PCR (b)	Ratio (b/a) %
Phenylalanine ammonia lyase (PAL)	*EguPAL_*	CT987001	EG__Ba	15	11	63
Cinnamate 4-hydroxylase (C4H)	*EguC4H*	CT988030	EG__Bb	6	2	33
4 coumarate CoA ligase (4CL)	*Egu4CL*	AJ244010	EG__Bb	15	12	80
Hydroxycinnamoyl-CoA:shikimate/quinate hydroxycinnamoyl transferase (HTC)	*EguHTC*	CT980202	EG_Ba	13	13	100
*p*-coumarate 3-hydroxylase (C3H)	*EguC3H*	CT986440	EG__Bb	5	3	60
Caffeoyl-CoA *O-*methyltransferase (CCoaOMT)	*EguCCoaOMT*	AF168778	EG_Ba	19	7	37
Cinnamoyl CoA reductase (CCR)	*EguCCR*	X79566	EG_Ba	13	10	77
Ferulate 5 hydroxylase/coniferaldehyde 5 hydroxylase (F5H/CAld5H)	*EguF5H*	CT987560	Eg_Ba	17	5	29
Caffeic acid/5- hydroxyoniferaldehyde *O*-methyltransferase (COMT)	*EguCOMT*	X74814	EG__Bb	19	12	63
Cinnamyl Alcohol dehydrogenase	*EguCAD2*	X65631	EG__Ba	22	13	59
Rho-related small GTP-binding protein	*EguRAC1*	DR410036	EG__Bb	33	3	9
R2R3 MYB transcription factor	*EguMyb1*	AJ576024	EB_Ba	10	9	90
R2R3 MYB transcription factor	*EguMyb2*	AJ576023	EG__Bb	7	6	86

Screening was made on subsets of EG_Ba and EG_Bb BAC libraries with redundant genome coverage of 8.5× and 7.5×, respectively. Number of positive clones obtained (a) and validated by PCR (b).

To remove false positives and target a single genetic locus, we performed an additional confirmation by PCR, using specific primer pairs designed from available *E. gunnii *available cDNA sequences. On average, the estimated proportions of 62% and 57% of the hybridization positive clones were confirmed by PCR screening for the EG_Ba and EG_Bb libraries, respectively (Table 1).

The results of the hybridization experiments compared to those obtained by PCR validation suggest that these genes may be present in duplicate or belong to multigene families present in the *Eucalyptus *genome, in agreement with the EST analysis of Rengel et al. [14] that found different unigene members for some of these genes.

### Sequencing of selected BAC clones

Five BAC clones were selected, sequenced and assembled with Roche GS FLX sequencing, and Newbler assembly methodology, respectively (Table 2). They included one BAC clone containing the chloroplast genome (EG_Ba_35H24), one BAC clone randomly selected from the EG_Ba library (EG_Ba_18G23), and three BAC clones (EG_Ba_2B15, EG_Ba_11K15, EG_Bb_94G18) that were hybridization positive for three genes of interest - *EguCCR, EguCAD2 and EguRAC1 *- respectively.

**Table 2:** Sequencing (454FLX) and assembly of selected BAC clones.

BAC	Gene/Probe	Reads	% in the assembly	% repeat	Scaffolds	Large Contigs (> 500Kb)	Observed lenght	Assembly length
**EG__Ba_2B15**	***EguCCR***	27,155	97.3	4.1	1	19	~145 kb	147,199
**EG__Ba_11K15**	***EguCAD2***	30,804	97.8	1.6	2	21	~130 kb	136,759
**EG__Bb_94G18**	***EguRAC1***	28,580	98	2.9	6	25	~120 kb	-
**EG__Ba_18G23**	***Randomly chosen***	29,837	97.7	0.9	4	24	~160 kb	174,430
**EG__Ba_35H24**	**Chloroplast**	31,081	98.3	1.72	1	4	~170 kb	160,137

The shotgun sequencing of these BAC clones produced on average 29,491 high quality reads per clone sequenced with a mean read length of 261nt. These clones were sequenced to different levels of sequence coverage ranging from 44.6× to 62.1×.

On average 98% of these reads were used to assemble the full sequences of each clone into a minimum of five and a maximum of 36 contigs, for EG_Ba_35H24 and EG_Ba_18G23, respectively. The number of large contigs (> 500 bp) varied among clones from 4 (for clone EG_Ba_35H24) to 25 (for clone EG_Bb_94G18). These contigs were then reassembled into one (clone EG_Ba_2B15) to six (clone EG_Bb_94G18) scaffolds, allowing the reconstruction of the full sequence of all BAC clones except clone EG_Bb_94G18 (6 scaffolds). In this latter case, the presence of repetitive sequences prevented our ability to order and orient four of the six. Such a problem was already reported in barley by Wicker et al. [45]. Despite the relatively restricted number of clones sequenced, our results suggest the feasibility of using 454 sequencing for rapid and cost-effective sequencing and assembly of *Eucalyptus *BAC clones. The increased length of the 454 reads currently achievable with the Titanium chemistry (expected size ~400-550 bp) should result in regions of high-quality finished genomic sequences. BAC clone sequences were deposited into GenBank (accession ID HM366540 to HM366544).

### Characterization of genomic nuclear BAC clone sequences

RepeatMasker http://www.repeatmasker.org/ was used to estimate GC content and search for interspersed repeats and low complexity DNA sequences in the four BAC sequences (588,509 bp, 13 scaffolds). The four BAC clone sequences revealed a low number of transposable elements, and low percentage of low complexity sequences (Additional file 1) when compared to 10 to 35% in other plant genomes analyzed (The Arabidopsis Genome Initiative 2000; International Rice Genome Sequencing Project 2005). However, these estimates cannot be generalized to the entire *E. grandis *genome due to the small number of BAC clones analyzed as well as the ascertainment bias resulting from the selection of gene-containing BACs. Furthermore, it is known that the distribution of these repetitive elements vary along the genomes. Indeed, clone EG_Ba_2B15 presented the lowest repeat content (1.2% of 152,083 bp) while the highest levels were found for clones EG_Bb_94G18 (5.5% of 129,018 bp) and clone EG_Ba_11K15 (5.4% of 137,697 bp). Six putative retroelements were found within the scaffold sequences of clone EG_Ba_18G23 (1.2% total length, 3 LINES and 3 LTRs) whereas none were identified in clone EG_Ba_2B15. Four LTR class retroelements were found within the scaffold sequences of clone EG_Bb_94G18 (2.5% total length, 3 Ty1/Copia and one Gypsy/DIRS1) and EG_Ba_11K15 (3,6% total length, 4 Gypsy/DIRS1). Low complexity sequences covered 0.8%, to 1.9% of the BAC clones sequence scaffolds analysed

Using Sputnik (Abajian, 1994, http://www.cbib.u-bordeaux2.fr/pise/sputnik.html) a total of 88 microsatellites also called simple sequence repeats (SSR) were found within the BAC scaffold sequences and these can be developed as new genetic markers. SSR markers have been extensively used in linkage analysis and comparative mapping of *Eucalyptus *species[10,46-48], genetic fingerprinting [49], population genetics [50], and for clonal fidelity assessment [51]. One possible application of these new BAC-derived genetic markers could be the anchoring of a physical map to the available *Eucalyptus *genetic maps. Within the BAC clone sequence scaffolds we found 57 perfect Class I SSRs, that are more likely to be polymorphic [52] as SSR mutations tend to be positively correlated with SSR length [53]. This class of SSRs was found to occur on average every 10.3 kb within the sequences of the four nuclear BAC clones sequenced, from a minimum of 7.2 kb [EG_Bb_94G18] to 15.3 kb [EG_Ba_11K15]. These differences could reflect the non-random distribution of SSR in plant genomes [52,54,55]. The frequency of ClassI SSRs observed in this study was similar to that observed by Mun et al. [54] for selected *Medicago truncatula *BAC clone sequences, but very low when compared to that observed in another tree species *Populus*, where SSRs occurred on average every 2.5 kb [55]. However, one cannot discard the possibility that the existence of gaps generated by the presence of repetitive sequences, where 454 sequencing has trouble going through, and/or due to a low sequence coverage in the region, might potentially underestimate the SSR frequency. Furthermore, the frequency of SSRs within the BAC clones is also very low when compared to that observed by Ceresini et al. [56]) and Rabello et al. [57] that reported a frequency of one Class I SSR every 2.5-2.7 kb in *Eucalyptus *cDNA libraries.

### Comparison of the structure and sequence of the CCR and CAD2 genes from *E. grandis *and *E. gunnii*

Cinnamoyl CoA reductase (CCR; EC 1.2.1.44), that catalyzes the conversion of cinnamoyl CoA esters to their corresponding cinnamaldehydes and Cinnamyl alcohol dehydrogenase (CAD; EC 1.1.195) that catalyzes the conversion of these aldehydes to the corresponding alcohols are considered key enzymes in lignin biosynthesis. For instance, it was previously found that CCR down-regulated plants had lower lignin levels than controls [58,59] and the extractability of the lignin polymer was improved in CAD down-regulated plants [60,61]. Sequences of positive BAC clones for *CAD *and *CCR *probes were analyzed for gene identification via homology searches. Searches with BLASTN performed against a non-redundant databases (e-value cut off of 1e^-5^) allowed us to easily identify the *E. grandis *homologous genes to the *E. gunnii CCR *gene (within scaffold #1 of EG_Ba_2B15) and the CAD2 gene (within scaffold#2 of clone EG_Ba_11K15).

**Figure 3:**
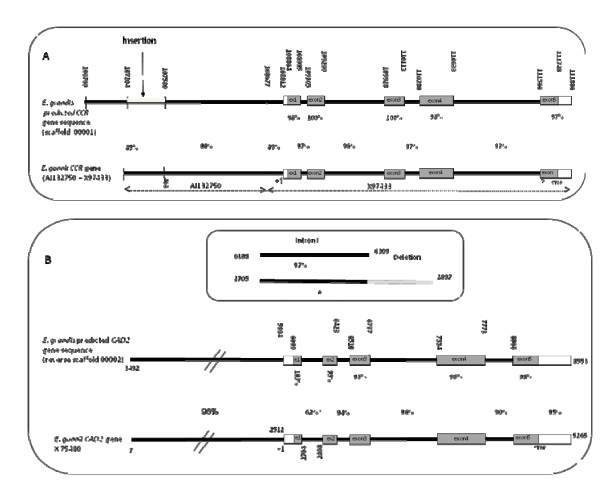
**Genomic structure comparison of the CCR (a) and the CAD2 (b) genomic clones between *E. grandis *and *E. gunnii***. Global alignement was performed by the Needle software (EMBOSS package). (a) The predicted *E. grandis *CCR genomic sequence found in scaffold #1 (108,677 to 111,886bp in Eg__Ba_2B15) was compared to the *E. gunnii *CCR promoter (EMBL AJ132750) linked to the genomic sequence (EMBL X97433). (b) The predicted *E. grandis *CAD genomic sequence found in scaffold #2 (3,492 to 8553bp in Eg__Ba_2B15) was compared to the *E. gunnii *CAD genomic sequence (EMBL X75480).

Global sequence alignments performed using the Needle algorithm included within the Emboss package [62] allowed for the calculation of the percent identity between *E. grandis *and *E. gunnii CCR *and *CAD *sequences and to compare their intron/exon structure. Results of these structural comparisons are schematically presented in Figure 3(a, b), and revealed that the number of exons, the intron/exon structure and junction boundaries were strictly conserved for both genes in both species. Moreover, the sequences are highly conserved particularly in the exons, with identity percentages varying between 98 and 100%. It seems that for both genes, the three first exons are slightly more conserved between the two species than the fourth and fifth exons.

Non-coding regions were also very well conserved between *E. gunnii and E. grandis CAD and CCR *sequences, respectively. The *CAD *promoter regions exhibited sequence identity of 96% for the first 2.5 kb. Whereas the sequence conservation was lower between the *CCR *promoter sequences, showing 88% sequence identity within the first 500 pb upstream the transcription start. The alignment was interrupted by an insertion of 444 bp in the *E. grandis *sequence and the identity level in the remaining 5' sequence dropped to 85%.

Concerning introns, identities between *E. grandis *and *E. gunnii *increased from 90% to 97%, the less conserved being intron 4, showing 90% and 93% identity for *CAD2 *and *CCR *respectively. Intron 1 in the *E. grandis CAD2 *gene exhibited a deletion of 71 nt after position 122 as compared to the corresponding intron in *E. gunnii*.

The indels, reported here, between the *CCR *promoters and between introns 1 of *CAD2 *could be used to develop markers to discriminate these two species, as they seem highly conserved within each species (Additional file 2).

### The *E. grandis *chloroplast genome characterization

**Figure 4:**
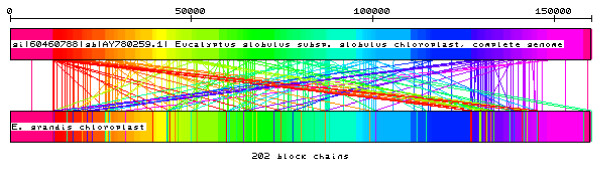
**CSA algorithm output showing the conserved sequences identified between the *E. grandis *(EMBL **HM347959**) and *E. globulus *(NC_008115) chloroplast genomes**.

The large size of *E. grandis *BAC clone inserts coupled with the library screening strategy (arrays hybridized with a pool of probes selected along the *E. globulus *chloroplast genome, NCBI accession number NC_008115) allowed for the identification of BAC clones with inserts that potentially contained the entire sequence of the *E. grandis *choloroplast genome. Clone EG_Ba_35H24 was selected for sequencing since this clone was shown to be positive for several chloroplast probes and also presented an insert size close to that of the previously sequence of *E. globulus *chloroplast genome (~160 kbp). The The Roche GS FLX reads were assembled into four long contigs sharing more than 99% sequence identity with the *E. globulus *chloroplast genome sequence. Due to the presence of inverted repeats (IRs) in the *E. globulus *chloroplast, a manual rearrangement of the sequences was performed based on this reference chloroplast genome [17] which allowed us to obtain a unique continuous fragment with 160,137 bp (EMBL HM347959), a length that is close to that observed for *E. globulus *(160,268 bp) [17] and *Vitis *(160,928 bp) [63] but larger than the one reported earlier for *E. nitens *(151 Kbp) based on restriction enzyme mapping [64]. The chloroplast genome of *E. grandis *includes a pair of inverted repeats 26,390 bp long, separated by a small, and large single copy regions of 18,478 bp and 88,879 bp, respectively. The GC-content of the *E. grandis *chloroplast genome is 36.9%, which is comparable to that of the *E. globulus *chloroplast genome and to other tree plant plastids (e.g. 38.5% in *Pinus thunbergii *[65], 36.7% in *Populus trichocarpa *[66], 37.4% in *Vitis vinifera *[63]). Figure 4 illustrates a very high conservation of the chloroplast genomes between the *E. grandis *and the *E. globulus*. Moreover, the annotation of the *E. grandis *chloroplast genome sequences reveals that gene order is conserved in the two species. The large size of the inserts in the BAC libraries allowed us to obtain the chloroplast genome sequence in one single BAC clone. The sequencing of this BAC clone with Roche GS FLX technology was as efficient as the bridging shotgun library strategy used for *E. globulus *[17] or *Vitis *[63] chloroplast genome sequencing. Such an approach could be readily applied to study other non-nuclear genomes, such as mitochondrial genomes. The complete and annotated *E. grandis *chloroplast genome sequence was deposited into GenBank (accession ID NC_014570).

## Conclusions

The two *Eucalyptus *BAC libraries described in this study represent an important milestone for the advancement of *Eucalyptus *genomics and forest tree research. These BAC resources have a highly redundant genome coverage (> 15×), contain large average inserts (157 and 135 kb) and have a very low percentage of clones with organellar DNA or empty vectors. This indicates that these publicly available BAC libraries are suitable for a broad range of applications in genetic and genomic research in *Eucalyptus *and possibly in related species of *Myrtaceae*, including genome sequencing, gene isolation, functional and comparative genomics. The analysis of ~0.6 Mb of BAC clone sequences generated by Roche GS FLX sequencing technology provided an overview of the composition of the *Eucalyptus *nuclear genome and the feasibility of using this high-throughput technology for low-cost and efficient sequencing and assembly of the targeted *Eucalyptus *sequences. SSRs identified within the BAC clone sequences could be used to develop new genetic markers for multiple genotyping purposes. In addition, we report the full chloroplast genome sequence of *E. grandis *(160,137 bp) allowing comparison of this genome with *E. globulus *and other plant species. Comparative analysis of the *CAD2 *and *CCR *genes between *E. grandis *and *E. gunnii *showed a high conservation of the structure of genes as well as a high identity both in the coding and non coding sequences.

## Methods

### Plant material and DNA preparation

High Molecular Weight (HMW) DNA was prepared from young leaves of clonally propagated plants of tree BRASUZ1 grown in partial shade at the Federal University of Viçosa, Brazil. BRASUZ1 is a S1 individual (one generation of selfing) of an elite *E. grandis *tree originally derived from seed lots collected in Coffs Harbor (Australia). BRASUZ1 was developed by Suzano Papel e Celulose Co. in 1987 and confirmed as S1 by microsatellite genotyping (D. Grattapaglia, unpublished). It is a fast growing tree that flowers normally and does not exhibit any sign of inbreeding depression. This tree is being used by the *Eucalyptus *Genome project currently under production at JGI (Joint Genome Institute) for its lower genomic heterozygosity as compared to a regular outcrossed tree to facilitate assembly. Tender, expanded leaves were collected during a time period of two months and kept frozen at -80°C. For each extraction, approximately 50 grams of frozen leaf tissue was ground to powder in liquid nitrogen with a mortar and pestle used to prepare megabase-size DNA embedded in agarose plugs as described by Zhang et al. [67] Agarose plugs containing high molecular weight (HMW) nuclei DNA were subsequently sent submerged in ethanol 95% to the Arizona Genomic Institute (AGI) for library construction.

### BAC library construction and BAC clone characterization

BAC libraries were constructed [68] using modifications recently described for *Oryza *[30]. DNA digestions were performed with 0.5 Unit *Hind*III in 100 μL reaction volume (EG_Ba library) and 0.8 Unit *Bst*YI also in 100 μL reaction volumeFor (EG_Bb libary) to obtain the appropriate partial digestion conditions. the EG_Ba BAC library, pulsed-field gel electrophoresis (PFGE) size-selected restriction fragments were ligated to the pAGIBAC1 vector (a modified pIndigoBAC536Blue with an additional *Swa*I site), while for the EG_Bb library, size-selected *BstY*I digested fragments were ligated to BamHI digested vector. Ligation products were transformed into DH10B T1 phage resistant *Escherichia coli *cells (Invitrogen, Carlsbad, CA) and plated on LB agar that contained chloramphenicol (12.5 μg.mL^-1^), X-gal (20 mg.mL^-1^) and IPTG (0.1 M). Clones were robotically picked into 384-well plates containing LB freezing media. Plates were incubated for 18 h, replicated and then frozen at -80°C for long term storage.

To estimate insert sizes, 5 μL aliquots of extracted BAC DNA were digested with 5 U of *Not*I enzyme for 3 hrs at 37°C. The digestion products were resolved by PFGE (CHEF-DRIII system, Bio-Rad) in a 1% agarose gel in TBE buffer 0.5×. Electrophoresis was carried out for 16 hours at 14°C with an initial switch time of 5 sec, a final switch time of 15 sec, in a voltage gradient of 6 V.cm^-1^. Insert sizes were compared to those of the MidRange I PFG Marker (New England Biolabs).

### BAC library screening

#### a) High Density Filter production and hybridization

A subset (plates 1-96) of libraries EG_Ba and EG_Bb was used for screening. High density colony filters for both libraries were prepared using a Genetix Q-bot (Genetix, New Milton, Hampshire, United Kingdom). Each 22.5×22.5 cm filter (Hybond-N+; Amersham Bioscience, Piscataway, NJ, USA) contained 36,864 independent clones arrayed in a double spotted 6×6 pattern. Spotted nylon membranes were transferred to solid LB agar plates supplemented with 12.5 μg/mL chloramphenicol, and bacterial clones allowed to grow overnight at 37°C, followed by transferred to 4°C just until colonies became confluent. Membranes were kept for 4 min onto a Whatmann 3 MM paper saturated with denaturation buffer (0.5 M NaOH, 1.5N NaCl), treated for 10 min at 100°C, and neutralized for 10 min on Whatmann 3 MM paper saturated with neutralization buffer (1.5 M Tris-HCL pH7.4, 1.5N NaCl). Immediately membranes were incubated at 37°C for 45 min with 250 mg/L proteinase K in 100 mM Tris-HCl pH 8, 50 mM EDTA, 0.5N NaCl. Finally, membranes were dried for 45 min at 80°C and UV-crosslinked (120,000 μJ.cm^-2 ^for 50 sec).

Labelling of organellar or nuclear probes was performed with [α-^33^P] dCTP (Perkin-Elmer, Waltham, Massachusetts, USA) on DNA (150 ng amplified DNA fragment) using the Ready-To-GO DNA Labeling kit. The dCTP (GE Healthcare, Waukesha, WI) and the non-incorporated nucleotides were removed using Illustra-ProbeQuant G50 (GE Healthcare, Waukesha, WI) according to manufacture's instructions.

Before performing hybridization, membranes were incubated for 30 min at 50°C in 6× SSC. Filters were pre-hybridized for 2 hours in 50 mL of hybridization buffer (6XSSC, 5X Denhardt, 0.5% SDS, 100 μg.mL^-1 ^denatured salmon sperm DNA) at 68°C. Hybridizations were carried out in high stringency conditions at 68°C overnight using 50 mL of fresh hybridization buffer supplemented with a minimum of 10^7^cpm purified and denatured probe per mL of buffer. Membranes were washed for 15 min at 50°C in SSC2x and SDS 0.1% buffer, followed by a second wash at 50°C for 30 min in SSC 0.5X and SDS 0.1% buffer. Finally, they were wrapped in a plastic film, exposed to the General Purpose PhosphorImager screen (Amersham Bioscience, Piscataway, NJ, USA) for a period of three days, and finally scanned using a Storm System (Amersham Bioscience (Amersham Bioscience, Piscataway, NJ, USA), set to a resolution of 50 μm.

#### b) Organellar DNA content estimation

To estimate the percentage of chloroplast and mitochondrial DNA content in each library, one high-density filter from each library was screened separately with a pool of five *E. globulus *probes for chloroplast genes *psbA, psbB, psbD, rbcL, ndhB *and with a pool of probes for mitochondrial genes *ccb256, ccb452, cox3 *[69]. Chloroplast probes were obtained by amplification of *E. globulus *DNA using specific chloroplast primers (Additional file 3). Positive clones for chloroplast and mitochondria probes were identified and 10% of these clones were used in the PCR validation step. BAC clone (EG_Ba_35H24] was selected to be full sequenced, and was subsequently sequenced by Roche GS FLX technology (454 Life Sciences, Branford, CT, USA) by Cogenics company(Grenoble, France).

#### c) Screening for selected genes

Each library was screened with different probes for genes of lignification biosynthesis pathway and for three regulatory genes (*EguRAC1; EguMyb1, EguMyb2*). Characteristics of each gene probe are described in Table 1. Clone inserts were amplified using universal primers M13 (5'CAGGAAACAGCTATGACC3') and M13 reverse (5'TGTAAAACGACGGCCAGT3'), and the identity of the amplicons checked by sequencing before use in the hybridization. Hybridization positive clones were then validated by PCR using specific gene primers (Additional file 4).

### BAC clones sequencing

Once selected, BAC clones were isolated, and the presence of the gene of interest was re-confirmed by PCR on isolated colony. BAC insert size was determined as described above. BAC DNA extractions, sequencing library generation, and sequencing were performed by Cogenics (Grenoble, France). BAC insert were sequenced by pyrosequencing using a Roche GS FLX Life Sciences instrument (Branford, CT, USA) [70,71]. The Newbler software (454 Life Sciences, Branford, CT, USA) was used to perform *de novo *assembly of the Roche GS FLX. 454 These assemblies may still contain indel errors even at high coverage.

### *E. grandis *chloroplast genome sequence annotation

The *E. grandis *chloroplast genome was annotated using the *E. globulus *chloroplast genome (NCBI accession id NC_008115) as the reference genome and following the annotation pipeline detailed in Addicional file 5.

Since these two genomes correspond to circular genomes, it was not guaranteed that the linear sequence obtained from sequencing was cut at the same genome position. An important step before using Multiple Sequence Alignment (MSA) algorithms on genome annotation of circular genomes includes the circularization and rotation of the genomes that are to be aligned. MSA algorithms were developed to deal with linear genomic sequences and in this sense they are very sensitive to the location where the genomic sequence begins. To improve the MSA alignment of both *E. grandis *and *E. globulus*, these two genomes were first processed using the CSA algorithm [72]. This algorithm identified the largest chain of non-repeated longest subsequences common to these two circular genomes. The genomes were then rotated and made linear for MSA purposes.

After the pre-processing step, the Dual Organellar GenoMe Annotator (DOGMA) software package [73] was used to perform BLAST searches against a custom database of plant chloroplast genomes.

To conclude the annotation procedure, the Artemis Comparison Tool (ACT) [74] was used. With this tool it was possible to visualize and solve any the remaining annotation inconsistencies, by comparing the annotation obtained from DOGMA with the *E. globulus *chloroplast genome sequence. Each gene structure was manually checked to define putative exons.

## Authors' contributions

JAPP participated in the conception of the study, in its design, and carried out all the experimental studies listed, and prepare the original draft of the manuscript. EP and SV participated in BAC macroarray preparation, hybridization and analysis. SB prepared HMW DNA agarose plugs. XS, JA, DK, RAW, produced the BAC libraries; MDS, PGSF, HSC and ATF participate in bioinformatics analysis and annotation of BAC clones. DG check the identity of BRASUZ1 and participated in the definition of BAC libraries. HB and JGP participated in the conception of the study, in its design. All authors read and approved the final manuscript.

## Supplementary Material

Additional file 1 **BAC sequences characteristics**. Click here for file

Additional file 2 **Analysis of polymorphism found at promoter of CCR gene and intron of CAD gene beetween the *E*. grandis (Transversaria section) and *E. gunnii *(Maidenaria section)**. Click here for file

Additional file 3 **Screening of the *E. grandis *BAC libraries for chloroplast and mitochondria genomes**. Primers used for PCR amplification of organelles specific probes. Click here for file

Additional file 4 **Table of primers used to validate hybridization positive clones**. Click here for file

Additional file 5 ***E. grandis *chloroplast genome annotation pipeline**. Click here for file
