# Supplementary material for: Advancing Eucalyptus genomics: identification and sequencing of lignin biosynthesis genes from deep-coverage BAC libraries
Source: BMC Genomics. 2011 Mar 4;12:137. doi: 10.1186/1471-2164-12-137 (PMC3060884; doi:10.1186/1471-2164-12-137)
Supplement: Additional file 1 — BAC sequences characteristics. [file 1471-2164-12-137-S1.PDF]

Additional file 1 -– BAC sequences characteristics. GC content, retro-elements, total interspersed repeats, simple repeats, low complexity DNA sequences.

	**EG__Ba_11K15 (*EguCAD2*)**			**EG__Ba_2B15 (*EguCCR*)**			**EG__Ba_18G23 (Randomly selected)**			**EG__Bb_94G18 (*EguRAC1*)**		
Sequences:	2			1			4			6		
Total length	137697			152083			169711			129018		
GC level	40,79			39,18			39,87			39,95		
bases masked:	7400			1833			5445			7062		
bases masked (%):	5,37			1,21			3,21			5,47		
	Number of elements	Length occupied	% of sequence	Number of elements	length occupied	% of sequence	Number of elements	length occupied	% of sequence	Number of elements	length occupied	% of sequence
Retroelements	4	4977	3,61	0	0	0	6	2024	1,19	4	3222	2,5
LINEs:	0	0	0	0	0	0	3	663	0,39	0	0	0
L1/CIN4	0	0	0	0	0	0	3	663	0,39	0	0	0
LTR elements	4	4977	3,61	0	0	0	3	1361	0,8	4	3222	2,5
Ty1/Copia				0	0	0	2	1084	0,64	3	3142	2,44
Gypsy/DIRS1	4	4977	3,61	0	0	0	1	277	0,16	1	80	0,06
Total interspersed repeats		4977	3,61	0	0	0		2024	1,19		3222	2,5
Low complexity	32	1540	1,12	27	1167	0,77	46	2450	1,44	41	2434	1,89
