# Supplementary material for: Advancing Eucalyptus genomics: identification and sequencing of lignin biosynthesis genes from deep-coverage BAC libraries
Source: BMC Genomics. 2011 Mar 4;12:137. doi: 10.1186/1471-2164-12-137 (PMC3060884; doi:10.1186/1471-2164-12-137)

Additional file 2 – Analysis of polymorphism found at promoter of CCR gene and intron of CAD gene beetween the *E.* grandis (Transversaria section) and *E. gunnii* (Maidenaria section).

Primer	Base on	Primer sequence	Initial position	Final position	Annealing temperature (ºC)
**CAD –F**	X75480.1 *E.gunnii* CAD gene	TTCGCTGTGCTTGTGATGTCGTCT	2762	2785	58.5
**CAD –R**	X75480.1 *E.gunnii* CAD gene	CTGTGGCAAATCCCGCAGCTC	2954	2934	58.7
**CCR-F**	BAC EG__Ba_2B15, scaffold00001	TGGACGTGAAGGATTTCCTGCCA	107146	107168	58.3
**CCR-R**	BAC EG__Ba_2B15, scaffold00001	GTGTGCGTAAGCAATCAAAGCCCT	107721	107698	58.5

Primers were designed to surround the polymorphic regions for both CAD and CCR genes, and performed PCR with DNA of six independent individuals for each species. As showed in the picture below, it was detected for both genes distinct and specific polymorphic bands between the two species. Cloned *E. gunnii* CAD gene and cloned *E. gunnii* CCR promoter was used as positive control for *E. gunnii* species. BAC clones EG__Ba_2B15 and EG__Ba_11K15 were used as positive controls for *E. grandis* species.

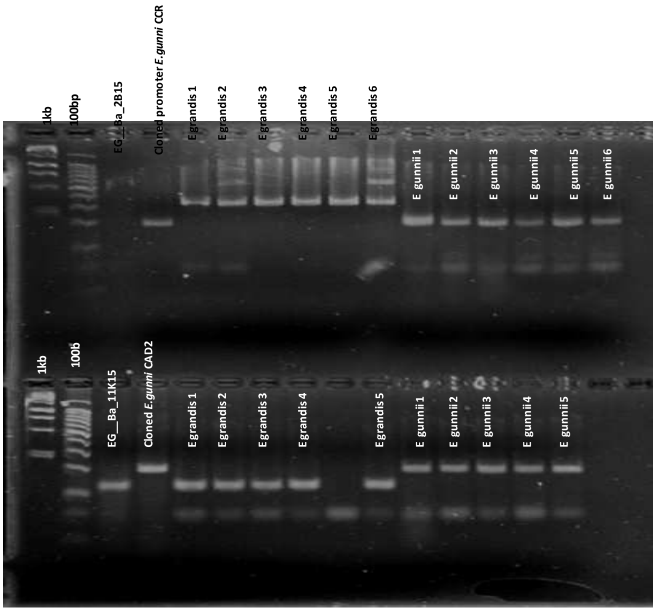

Supplement: Additional file 2 — Analysis of polymorphism found at promoter of CCR gene and intron of CAD gene beetween the E. grandis (Transversaria section) and E. gunnii (Maidenaria section). [file 1471-2164-12-137-S2.PDF]
