# Supplementary material for: Advancing Eucalyptus genomics: identification and sequencing of lignin biosynthesis genes from deep-coverage BAC libraries
Source: BMC Genomics. 2011 Mar 4;12:137. doi: 10.1186/1471-2164-12-137 (PMC3060884; doi:10.1186/1471-2164-12-137)
Supplement: Additional file 3 — Screening of the E. grandis BAC libraries for chloroplast and mitochondria genomes. Primers used for PCR amplification of organelles specific probes. [file 1471-2164-12-137-S3.PDF]

Additional file 3 - Screening of the *E. grandis* BAC libraries for chloroplast and mitochondria genomes. Primers used for PCR amplification of organelles specific probes.

Genome	Gene	Forward primer	Reverse primer	Reference
Chloroplast	*psbA*	TACCATCCGAAAAACTTCCTTG	ACCTTATTGACCGCAACTTCTG	this article
	psbB	GAGATAAAGAAGGGCGTGAACT	ATTCCACTTGAGCATCCAAATC	this article
	psbD	CGCAGTTTCTACTCCCGCTAA	GCACCATCACCATCTTCAAATAA	this article
	rbcL	AATGATGAAAAGGGCTGTATTTG	CCAAGGGTGTCCTAAAGTTCCT	this article
	ndhB	TTTTATGCGGTGCTAACGATTT	TTGGTCCGAGTGGGGATAG	this article
Mitochondria	*ccb256*	GGAAGTTAGCAAAGTTAGAC	TTGTTCTTAACAGCGATGGC	[67]
	ccb452	TATTACCAGACATAATTGGG	TGAACTGTATCTTCTTTGTG	[67]
	cox3	CCGTAGGAGGTGTGATGT	CTCCCCACCAATAGATAGAG	[67]
