# Supplementary material for: Advancing Eucalyptus genomics: identification and sequencing of lignin biosynthesis genes from deep-coverage BAC libraries
Source: BMC Genomics. 2011 Mar 4;12:137. doi: 10.1186/1471-2164-12-137 (PMC3060884; doi:10.1186/1471-2164-12-137)
Supplement: Additional file 4 — Table of primers used to validate hybridization positive clones. [file 1471-2164-12-137-S4.PDF]

Additional file 4 - Table of primers used to validate hybridization positive clones

Gene family	Gene	EMBL accession	Foward	Reverse	Reference
Phenylalanine ammonia lyase (PAL)	*EguPAL_*	CT987001	GTGCAGGTCGTACCCATTGT	GGCAGTCTTCAGGATTTGGA	This article
Cinnamate 4-hydroxylase (C4H)	*EguC4H*	CT988030	CGGAAGAGATGAAGGTCGAG	AGACGATGGTGGAGTGCTTC	This article
4 coumarate CoA ligase (4CL)	*Egu4CL*	AJ244010	GTTGTGCCGATGAAGGATG	TGAAAAAGAAAAGGGTATGAGAAA	This article
Hydroxycinnamoyl-CoA:shikimate/ quinate hydroxycinnamoyl transferase (HTC)	*EguHTC*	CT980202	CGATCTATCAGCCCTTGTCC	CTTCATACGCAATTCCACCA	This article
p-coumarate 3-hydroxylase (C3H)	*EguC3H*	CT986440	TGGAAGTTTGTCATCGTCGT	CATATAAGGATCGCCTTTGGA	This article
Caffeoyl-CoA *O*-methyltransferase (CCoaOMT)	*EguCCoaOMT*	AF168778	CGC TCC ACC CCT TCC T	GGC TCC TTC ACG ACC TTT C	[72]
Cinnamoyl CoA reductase (CCR)	*EguCCR1*	X79566	TGATGAGGTGAACCCAAGAGTA	TTTCTCCTGCAAGCTCTTGACA	[15]
Ferulate 5 hydroxylase/ coniferaldehyde 5 hydroxylase (F5H/CAld5H)	*EguF5H*	CT987560	CGTCCCTCTTCTCCTCTTCC	GCCATACTTCTTCGCCAGAC	This article
Caffeic acid/5-hydroxyconiferaldehyde *O*-methyltransferase (COMT)	*EguCOMT*	X74814	CGC TCC ACC CCT TCC T	GGC TCC TTC ACG ACC TTT C	[72]
Cinnamyl Alcohol dehydrogenase	*EguCAD2*	X65631	AGGAAACAGAGGAGATGCTTGA	CTCTCTAGGGCGGTGTTGA	[15]
Rho-related small GTP-binding protein	*EguRAC1*	DR410036	AGCGCGTCGAGGTTCATCAA	TGGGGAAAGTGTTGCTGGTATA	[14]
R2R3 MYB transcription factor	*EguMyb1*	AJ576024	TCAATTACCTGCGGCCGGACC	TATAATCCAAAACACTTGCC	[73]
R2R3 MYB transcription factor	*EguMyb2*	AJ576023	TTAATTACTTGAGGCCCGACC	ATACTGACCGGTGCAATGCC	[74]
