# Supplementary material for: Advancing Eucalyptus genomics: identification and sequencing of lignin biosynthesis genes from deep-coverage BAC libraries
Source: BMC Genomics. 2011 Mar 4;12:137. doi: 10.1186/1471-2164-12-137 (PMC3060884; doi:10.1186/1471-2164-12-137)

Additional file 5 - *E. grandis* chloroplast genome annotation pipeline.

This pipeline includes the CSA algorithm to find the best rotation between the circular chloroplast genomes of *E. grandis* and *E. globulus*, the DOGMA software package that automates the annotation of organelle genomes and the Artemis software that was used to visualize and conclude the annotation procedure.

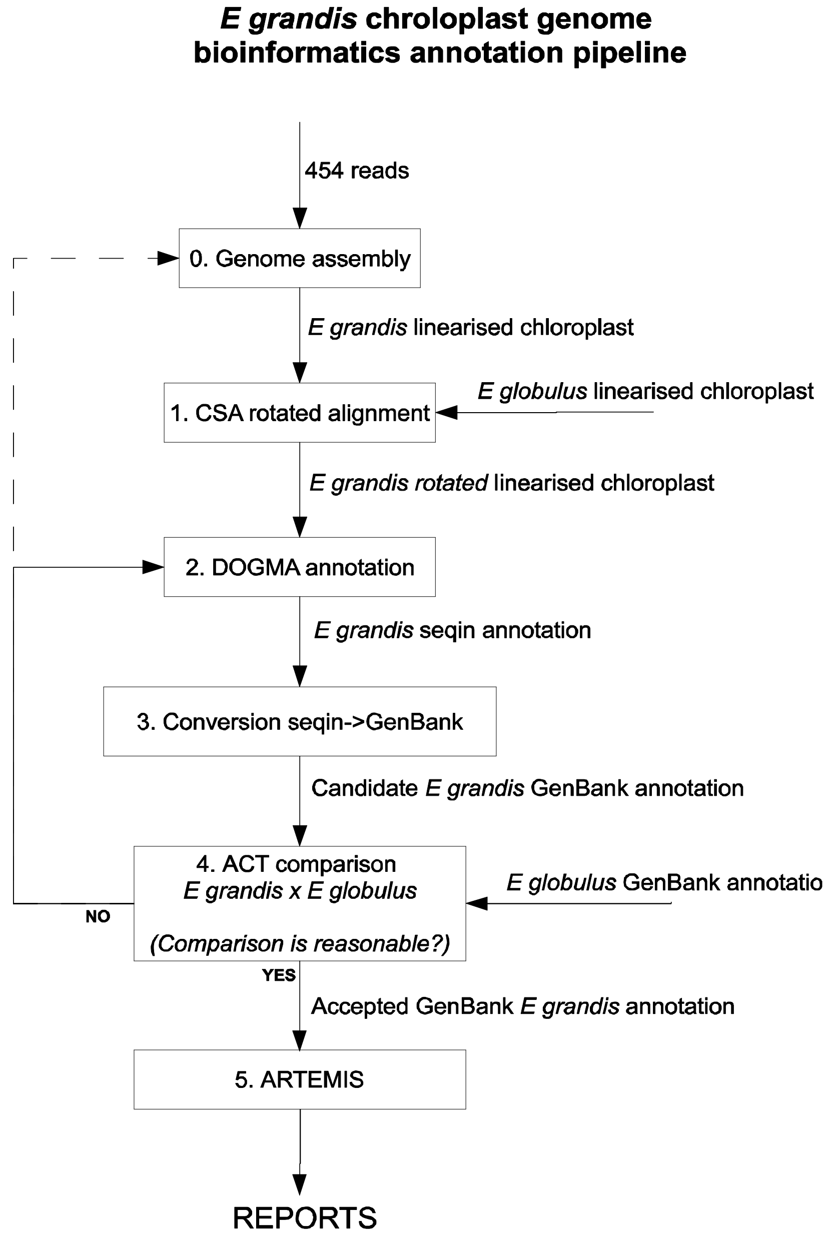

Supplement: Additional file 5 — E. grandis chloroplast genome annotation pipeline. [file 1471-2164-12-137-S5.PDF]
